# Can implicit associations and approach-avoidance tendencies be observed in both problematic social network use and tobacco use disorder? Evidence from a laboratory study

**DOI:** 10.1016/j.abrep.2026.100697

**Published:** 2026-04-15

**Authors:** Lasse David Schmidt, Lena Klein, Christian Montag, Hans-Jürgen Rumpf, Elisa Wegmann

**Affiliations:** a Department of Psychiatry and Psychotherapy, University of Lübeck, Lübeck, Germany; b General Psychology: Cognition, University of Duisburg-Essen, Duisburg, Germany; c Centre for Cognitive and Brain Sciences, Institute of Collaborative Innovation, University of Macau, Macau SAR, China; d Department of Computer and Information Science, Faculty of Science and Technology, University of Macau, Macau SAR, China; e Department of Psychology, Faculty of Social Sciences, University of Macau, Macau SAR, China; f Center for Behavioral Addiction Research (CeBAR), Center for Translational Neuro- and Behavioral Sciences (C-TNBS), University Hospital Essen, University of Duisburg-Essen, Essen, Germany

**Keywords:** Problematic social network use, Social media addiction, Tobacco use disorder, Implicit cognitions

## Abstract

**Background:**

Problematic social network use (PSNU) has gained increasing attention as a potential disorder due to addictive behavior. However, evidence regarding underlying cognitive mechanisms remains inconsistent. Implicit cognitive processes, such as implicit associations and approach-avoidance tendencies, have been proposed as relevant factors in substance use disorders and behavioral addictions, but their role in PSNU is still unclear. Given similarities in usage behavior between PSNU and tobacco use disorder (TUD), a direct comparison may help to identify converging and diverging mechanisms.

**Methods:**

In a laboratory study, a sample of N = 178 participants was classified into four groups using a structured diagnostic interview: individuals with PSNU (*n* = 53), risky use (*n* = 61), non-problematic use (*n* = 48), and TUD (*n* = 16). Implicit cognitions were assessed using the Implicit Association Test (IAT) and the Approach-Avoidance Task (AAT) with behavior-specific stimuli. Group differences were analyzed using ANOVA and ANCOVA, the latter statistical approach controlling for age and psychopathological symptoms.

**Results:**

Significant group differences emerged for the IAT, in individuals with PSNU and risky use showing more positive implicit associations towards behavior-related stimuli than the TUD group but did not remain stable when controlling for age and comorbid psychopathological symptoms. No significant group differences were found for approach-avoidance tendencies across all groups.

**Conclusion:**

Our results suggest that implicit cognitions may play a more complex role in PSNU than previously assumed. Implicit associations may already occur in early stages of addictive behaviors and approach-avoidance tendencies may depend on contextual and intraindividual factors. Future research should further address these factors, also by using advanced ecologically valid methods.

## Highlights

• Diagnostic interview grouped subjects into PSNU, risky, control and TUD groups.
• PSNU and risky group showed higher positive implicit associations (IAT) than TUD group.
• IAT differences were not robust after controlling for age and psychopathology.
• No group differences in approach-avoidance tendencies.
• Implicit cognitions in PSNU may emerge early and be context-dependent.

## 1 Introduction

Social network applications such as Instagram or TikTok offer their users a broad variety of possibilities for sharing information and social exchange (Wegmann and Brand, 2016, Wegmann and Brand, 2019) and, by that, play an important role in fulfilling various social needs (Bailey et al., 2020, Khang et al., 2014, Lee et al., 2011, Nadkarni and Hofmann, 2011). Consequently, the number of users of social networks (SNS) worldwide has increased significantly in recent years (Statista, 2025). However, despite positive aspects that using social networks may entail, growing evidence suggests that there is a substantial number of individuals that experience a problematic usage characterized by a loss of control on the usage behavior (Moretta & Wegmann, 2025), which is associated with significant negative consequences in their everyday life such as psychological distress and insomnia (Chen et al., 2020, Pagano et al., 2023) as well as psychopathological symptoms (Huang, 2022, Wang et al., 2022).

The inclusion of gaming disorder as disorder due to addictive behavior (Code 6C51) in the International Classification System of Diseases (ICD-11) of the World Health Organization served as a catalyst for the debate on whether and to what extent other excessive online behaviors should be considered as addictive behaviors as well (Brand, Rumpf et al., 2022). A growing number of researchers argue that the problematic use of social networks (PSNU) should be taken into account and potentially classified in the category “other specified disorders due to addictive behaviors” (ICD-11 Code: 6C5Y; Brand, Rumpf et al., 2022). At the same time. It should be noted that different terminological and conceptual approaches exist in the literature. For example, some researchers refer to the phenomenon as social networks use disorder (SNUD; Rumpf et al., 2025) or addictive social media use (e.g., Brailovskaia, 2024). However, since PSNU has not yet been formally included in the current classification systems as a distinct disorder, we use the more cautious label “PSNU” to emphasize the need for further empirical evidence for a future classification of PSNU as an addictive behavior (Moretta & Wegmann, 2025). Finally, we are aware of the fact that PSNU could also comprise other behaviors such as cyberbullying on the platforms. Here, we explicity mention that PSNU is studied within an addiction framework. Consequently, if PSNU could be characterized by addictive behavior patterns, a better understanding of the underlying affective and cognitive processes involved in the development and maintenance of this behavior is necessary. Thereby a direct comparison with other addictive behaviors or substance use disorders might be helpful for investigating the proximity to the addiction framework (Brand et al., 2021, Brand et al., 2022).

Dual-process models of addiction propose that addictive behaviors arise from an imbalance between impulsive, cue-driven processes and reflective control mechanisms (Stacy and Wiers, 2010, Strack and Deutsch, 2004). Within this framework, implicit cognitive processes such as automatic associations and approach-avoidance tendencies toward addiction-related stimuli are assumed to contribute to the development and maintenance of addictive behaviors. Building on this perspective, the Interaction of Person-Affect-Cognition-Execution (I-PACE) model by Brand et al. (2019) provides a comprehensive framework for understanding the development and maintenance of behavioral addictions and problematic usage of the internet in particular. The model assumes that interactions between predisposing variables (e.g., personality, psychopathology), affective and cognitive responses (e.g., cue-reactivity, craving, and implicit cognitions), and executive functions contribute to the initiation and continuation of addictive behaviors over time. Repeated reinforcement processes and learning mechanisms are assumed to strengthen cue-reactivity and craving, which in turn bias decision-making toward the execution of the behavior. Within this framework, implicit cognitive processes such as automatic associations and approach-avoidance tendencies are considered to play a crucial role, especially in later stages of the addiction process, when the behavior is conducted habitualized (Brand et al., 2016, Brand et al., 2019, Glock and Pit Ten-Cate, 2019).

Empirical research has demonstrated positive implicit associations and approach biases towards addiction related stimuli to be present in individuals with substance use disorders (Stacy & Wiers, 2010). Meta-analytic findings suggest the presence of positive implicit associations towards substance-related stimuli to be present in several substance use disorders, including alcohol use disorder and tobacco use disorder (TUD, Rooke et al., 2008). In terms of approach-avoidance tendencies, reviews suggest approach biases to occur across several substance use disorders, to be more pronounced in clinical populations than in healthy controls and to be linked to consumption behavior (Kakoschke et al., 2019, Loijen et al., 2020). However, opposite effects have also been observed: Avoidance of addiction-related stimuli was shown in individuals with alcohol use disorder undergoing treatment and was associated with a perceived loss of control (Townshend & Duka, 2007).

Research on behavioral addictions has produced more heterogeneous findings and many studies in this field are based on subclinical or analog samples. Positive implicit associations towards behavior-related stimuli have been previously identified in individuals with non-clinical problem gambling behavior (Brevers et al., 2013). Snagowski et al. (2015) outlined positive implicit associations toward pornographic stimuli in individuals with higher levels of problematic pornography use, which were linked to elevated levels of subjective craving in a general population sample. These findings could be replicated in a clinical sample of males with compulsive sexual behavior disorder (CSBD) by Engel et al. (2024). Further studies found significant correlations between implicit associations and buying-shopping disorder (Trotzke et al., 2020) as well as self-report measures of problematic internet use in general (Chen et al., 2018, Roh et al., 2018). Evidence regarding approach-avoidance tendencies suggests that individuals with gaming disorder exhibit stronger approach tendencies toward gaming-related cues (He et al., 2021, Li et al., 2024). This tendency has also been replicated in more ecologically valid paradigms, such as virtual reality–based AATs (Wei et al., 2023). Again, investigating a clinical sample, Engel et al. (2025) detected a stronger approach bias towards pornographic stimuli in males with CSBD. In addition, Snagowski and Brand (2015) found a curve-linear associations between problematic pornography use and approach-avoidance tendencies and consequently argue that both approach and avoidance reactions could be possible upon confrontation with addiction-related stimuli in individuals with behavioral addictions (Snagowski & Brand, 2015).

Concerning directly PSNU (for a detailed overview on implicit cognitions in PSNU see also Kessling et al., 2023), two studies found positive implicit associations related with symptom severity (Brailovskaia and Teichert, 2020, Turel and Serenko, 2020), while a more recent study found no significant differences between individuals with PSNU and healthy controls (Kessling et al., 2025). Regarding approach-avoidance tendencies one study found the relative level of PSNU to be associated with an implicit approach motivation for social network stimuli (Juergensen & Leckfor, 2019), while another study found approach tendencies only be linked to the frequency of checking social networks (Wadsley & Ihssen, 2022).

Taken together, the heterogeneity of results and methodological approaches in the field of behavioral addictions in general and PSNU in specific underlines the need for further research on the role of implicit associations and approach-avoidance tendencies. Consequently, direct comparison between PSNU and substance use disorders can provide valuable insights into shared and distinct cognitive mechanisms underlying addictive behaviors. Although direct empirical comparisons between both behaviors remain scarce, existing evidence points to meaningful associations between these domains. Both TUD and problematic online behaviors have been consistently linked to shared core mechanisms of addiction. In particular, cue-reactivity and craving are considered central processes in the development and maintenance of substance use disorders, including TUD (Betts et al., 2021), and are also discussed as key mechanisms in problematic use of the internet and PSNU (Antons et al., 2025, Wegmann et al., 2018). From a behavioral perspective, both tobacco use and social networks use are characterized by frequent and short interruptions of everyday life that may negatively affect productivity (Berg et al., 2019, Koessmeier and Büttner, 2021, Shiffman et al., 2014, Siebers et al., 2022) and reward-related anticipatory processes, possibly linked to dopamine (Liu et al., 2022, Westbrook et al., 2021). Given these parallels regarding the underlying mechanisms of both behaviors, a direct comparison in terms of implicit processes seems even more important, also to determine whether these represent distinct clinical conditions.

## 2 Objectives

Our present study aimed to investigate implicit associations and approach-avoidance tendencies in individuals with PSNU by directly comparing them with individuals showing risky social network use, which we defined as a stage in which negative consequences are already apparent, but not all criteria for a pathological disorder are met, non-problematic social network use and individuals with TUD. Participants were classified using a structured diagnostic interview based on adapted DSM-5 criteria for gaming disorder. By including different stages of social network use and a substance-related comparison group, our study seeks to contribute to a more differentiated understanding of the role of implicit cognitions in PSNU.

Thereby, we assume positive implicit associations and approach-tendencies towards specific behavior-associated stimuli to be strongest in individuals with addictive behaviors, resulting in the following hypotheses:

• H1: Individuals with PSNU and individuals with TUD show the highest positive implicit associations towards behavior-specific addiction-related stimuli, followed by individuals with risky SNS use and individuals with non-problematic SNS use.
• H2: Individuals with PSNU and individuals with TUD show the strongest approach-tendencies towards addiction related stimuli, followed by individuals with risky SNS use and individuals with non-problematic SNS use.

To account for the previous described inconsistencies in the research field, with some studies also illustrating the occurrence of avoidance tendencies in individuals with addictive behaviors and following the study by Snagowski and Brand (2015) on problematic pornography use, we conduct exploratory tests for any quadratic relationships between approach-avoidance tendencies and symptom severity of PSNU.

## 3 Method

### 3.1 Procedure and sample

The study was conducted as part of a preregistered multi-site research group (Brand et al., 2021, Wegmann et al., 2021; FOR2974; Preregistration of research group: https://osf.io/n5cd7/overview), at the universities of Lübeck and Duisburg-Essen in Germany. Participants were recruited between April 2022 and August 2024 via flyers, online advertisements, press releases, and a pre-screening website (see also Montag et al., 2023). Eligibility was assessed by a telephone-screening, assessing use of social networks, smoking behavior, and exclusion criteria (see below).

In order to confirm classification of individuals into the respective groups, an adapted version of the Structured Diagnostic Interview for Internet-Related Disorders (AICA-SKI:IBS; Müller et al., 2017) was conducted in the laboratory setting. The interviews were conducted by doctoral students in psychology, neuroscience, or medicine, who had received diagnostic training and were regularly supervised by experienced clinicians. Across all diagnostic steps of this study, the screening procedure, as well as the latter diagnostic interview, PSNU was assumed from five or more DSM-5 criteria fulfilled. A risky use was assumed from two to four criteria. All individuals fulfilling one or less criteria were defined as (non-problematic) control participants. To be included in the TUD group, individuals had to show neither problematic nor risky SNS use and a score higher than or equal to four in the Fagerström Test for Nicotine Dependence (FTND; Heatherton et al., 1991).

Inclusion criteria for all groups were age ≥16 and ≤65 years and sufficient German language skills. Exclusion criteria for all groups were current diagnosis of learning or developmental disorders, psychosis, mania, current substance-use disorder, acute suicidal ideation, and any psychoactive substances known to interfere with performance in cognitive tasks. All inclusion and exclusion criteria were addressed during screening procedure. Group classification and core inclusion criteria related to PSNU and TUD were further verified using the structured clinical interview as described above. All participants provided written informed consent and received financial compensation of €10 per hour of testing.

A total sample of 190 participants was recruited. After excluding cases with missing data or careless responding (as measured by self-report), the final sample consisted of *n* = 178 individuals (35% male; age *M* = 29.19, *SD* = 10.25 years, range = 16–65), including 53 participants with PSNU, 61 with risky SNS usage, 48 with non-problematic use of SNS and 16 participants with TUD. Table 1 shows the descriptive statistics of all subgroups of the sample and the statistical group comparisons. Group differences in sociodemographic variables (age, gender, education, and employment status) were examined using ANOVA and chi-square tests. Significant differences were found for age, education, and employment status, with the TUD group being significantly older than any of the social network groups (all *p* values ≤ 0.023) whilst showing the highest employment rates and the lowest educational level. No differences emerged for gender or relationship status (see Table 1) Further significant group differences were found regarding reported symptoms of depression, with the group of individuals with PSNU showing significantly higher expressions than the group of individuals with TUD (*p* = 0.009), and obsession-compulsion, with the PSNU group showing significantly higher expressions than any other group (all *p* values < 0.001; see Table 1).

**Table 1:** Descriptive statistics and comparisons of all subgroups of the sample.

	Social networks groups									TUD (*n* = 16)				comparison					
Measure	PSNU (*n* = 53)			Risky (*n* = 61)			Control (*n* = 48)												
	*Mean*	*SD*	*Range*	*Mean*	*SD*	*Range*	*Mean*	*SD*	*Range*	*Mean*	*SD*	*Range*		Test	*F*	*df1*	*df2*	*p*	*η2*
Age	25.98	6.67	17–58	27.87	9.19	18–62	30.35	11.10	16–64	41.31	12.54	25–65		Welch-ANOVA	8.16	3	56.09	<0.001	0.17
*BSI scales*																			
depression	1.08	0.90	0.00–3.60	0.83	0.76	0.00–3.20	0.67	0.93	0.00–3.80	0.31	0.47	0.00–1.60		ANOVA	4.26	3	174	0.006	0.07
anxiety	0.77	0.51	0.00–2.17	0.52	0.42	0.00–1.67	0.63	0.65	0.00–2.33	0.58	0.57	0.00–1.83		Welch-ANOVA	2.62	3	57.49	0.059	0.04
obsession-compulsion	1.51	0.77	0.00–3.33	1.03	0.66	0.00–2.83	0.77	0.62	0.00–2.67	0.59	0.38	0.00–1.33		Welch-ANOVA	15.47	3	70.28	<0.001	0.19

	*N*	%		*N*	%		*N*	%		*N*	%				*χ^2^*(3)			*p*	
*Sex*																			
male	16	30.2		22	36.1		16	33.3		9	56.3				3.77			0.287	
female	37	69.8		39	63.9		32	66.7		7	43.8								
Education: Qualification for university entrance	37	69.8		49	80.3		36	75.0		6	37.5				11.86			0.008	
Employed full-time or part-time	11	20.8		20	32.8		23	47.9		8	50.0				9.98			0.019	
Born in Germany	49	92.5		59	96.7		48	100.0		16	100.0				5.07			0.167	
In a relationship	31	58.5		29	47.5		25	52.1		8	50.0				1.402			0.705	

*Note.* PSNU = problematic use of social networks; TUD = tobacco use disorder; BSI = brief symptom inventory.

### 3.2 Ethics

All procedures were carried out in accordance with the Declaration of Helsinki. Ethical approval was obtained from the responsible institutional ethics committees at the University of Luebeck (ID: 20-081) and the University of Duisburg-Essen (ID of overall project: 1911APBM0457). To ensure pseudonymization of participant data and to comply with the European Union’s General Data Protection Regulation, we employed the encryption-based pseudonymization framework ALIIAS (Englert et al., 2023).

### 3.3 Implicit Association Test (IAT)

A modified version of the IAT (Greenwald et al., 1998) was used to assess implicit associations towards addiction-related pictures. The procedure and implementation of the IAT was carried out in line with preliminary studies in this field (Kessling et al., 2025, Snagowski et al., 2015). We used distal social network cues as target stimuli (“addiction-related”) for the respective groups of social network users (PSNU, risky, non-problematic; for a more detailed descriptions of the cues see Antons et al., 2025) These showed, from a first-person perspective, a person holding a device on which an Internet site was called up. The Internet sites for the social network cues showed log-in pages or starting-pages of web-representations of twelve social networking sites (e.g. TikTok, Instagram, Facebook, Snapchat, WhatsApp). For the TUD group, we used distal smoking pictures as target cues showing also from a first-person perspective a person holding smoking related devices such as ashtrays or lighters. The stimuli were semi-individualized for each subject based on gender and, in the case of social network stimuli, the two devices they primarily used (e.g., smartphone and tablet). For the attribute concepts, “positive” and “negative” pictures from the International Affective Picture System (Lang et al., 1997) were used. Smoking-related images served as non-addiction-related stimuli in the social network groups and vice versa in TUD group. Reaction time data was analyzed using the D2_SD_ algorithm (Greenwald et al., 2003), with higher scores indicating stronger positive implicit associations toward addiction-related stimuli. Further procedural details and stimulus examples are available in the project’s OSF repository (https://osf.io/9j8g3/overview).

### 3.4 Approach-Avoidance-Task (AAT)

The AAT (Rinck & Becker, 2007) was used to assess approach-avoidance tendencies towards addiction-related stimuli, as implemented in a previous study (Snagowski & Brand, 2015). Participants responded to addiction-related and neutral stimuli by pushing or pulling a joystick, corresponding to approach or avoidance movements. Addiction-related stimuli were utilized as in the IAT procedure, while neutral cues depicted, from a first-person perspective, everyday objects, for example pens or staplers, that could again be individualized according to the gender.

In line with previous works (Rinck and Becker, 2007, Snagowski and Brand, 2015), compatibility effect scores were calculated by subtracting median pull reaction times from median push reaction times for target and neutral stimuli separately. An overall-reaction-time (RT) score was computed to capture relative processing speed for addiction-related compared to neutral stimuli, with negative values indicating faster responses to target cues. Further technical details regarding the AAT-Procedure can be found on the OSF repository (https://osf.io/9j8g3/overview).

### 3.5 Self-report measures

Psychological distress was assessed using selected subscales of the *Brief Symptom Inventory* (BSI; Derogatis & Melisaratos, 1983), a 53-item self-report questionnaire designed to measure current psychological symptomatology. Specifically, the subscales *obsessive*–*compulsive* (6 items, *α* = 0.82, example: “Having to check and double-check what you do”), *depression* (5 items, *α* = 0.86, example: “Feeling no interest in things”), and *anxiety* (6 items, *α* = 0.75, example: “Feeling tense or keyed up”) were administered. Each item is rated on a 5-point Likert scale ranging from 0 (“not at all”) to 4 (“extremely”), indicating the degree of distress experienced during the past week. Scale scores were calculated as mean values, with higher scores indicating greater symptom severity.

Nicotine dependence in the subgroup of smokers was assessed using the *Fagerström Test for Nicotine Dependence* (FTND; Heatherton et al., 1991), a 6-item self-report measure assessing the intensity of physical nicotine dependence. Items cover aspects such as daily cigarette consumption, difficulties refraining from smoking, and time to the first cigarette after waking (example: “How soon after waking do you smoke your first cigarette?”). *Total scores* range from 0 to 10, with higher scores indicating greater nicotine dependence.

Symptom severity of PSNU was assessed using the *Assessment of Criteria for Specific Internet-use Disorders* (ACSID-11; Müller et al., 2022). The ACSID-11 is a self-report questionnaire developed to assess criteria for specific Internet-use disorders based on the ICD-11 framework for gaming disorder. It includes items assessing impaired control (example: “How often do you find it difficult to control your use of social networks?”), increasing priority given to the behavior, continuation despite negative consequences, and functional impairment. Items were answered with respect to social network use and rated on two 4-point Likert scales: (1) *frequency* (0 = “never” to 3 = “often”) and (2) *intensity* (0 = “not at all intense” to 3 = “intense”). Sum scores were computed separately for frequency and intensity to represent different aspects of symptom severity. The frequency score reflects how often problematic behaviors occur, whereas the intensity score captures the subjective level of distress and impairment. Internal consistency was excellent for both the frequency (*α* = 0.92) and intensity (*α* = 0.90) scales.

### 3.6 Statistical analysis

All statistical analyses were conducted using SPSS statistics (Version 29.0). We used analyses of variance (ANOVA) with group (PSNU group, risky group, control group, TUD group) as between-subject factor for testing our hypotheses. To complement the primary analyses and account for the limited sample size of the TUD group, additional analyses were conducted including only the three social network use groups. Bonferroni post-hoc tests were conducted to identify specific group differences. In the event of lack of variance homogeneity, Welch's ANOVA and Games-Howell post hoc tests were interpreted. We used partial *η^2^* to classify the effect sizes. According to the guidelines of Cohen (2013) the interpretation values were at 0.01 for small and at 0.06 for medium effects. The results were controlled for age and BSI-subscales of depression, anxiety, and obsession/compulsion as covariates. A chi-square test of independence was used to analyze associations between group assignments and categorical variables. Pearson correlations were utilized to describe associations between implicit task scores and psychopathological variables. Further exploratory analyses investigating potential linear or quadratic relations between AAT target compatibility effect score and ACSID-11 scores were conducted by means of linear and curve-linear regressions.

## 4 Results

### 4.1 Descriptive statistics and bivariate correlations

Descriptive statistics are shown in Table 1. Bivariate comparisons of implicit measures with selected psychopathological symptoms and behavioral measures, as illustrated in Table 2, revealed no significant correlations.

**Table 2:** Bivariate correlations between the implicit scores and selected variables.

Measure	Mean	SD	[1]	[2]	[3]	[4]	[5]	[6]	[7]	[8]
[1] IAT-D2_SD_ score	0.70	0.52	−							
[2] AAT Target compatibility effect Score	−128.44	88.60	0.178*	−						
[3] AAT Neutral compatibility effect Score	−184.86	109.98	−0.063	−0.423**	−					
[4] AAT Overall-RT Score	−34.93	41.86	0.157*	−0.179*	0.067	−				
[5] BSI-Depression	0.81	0.85	0.144	0.139	−0.070	−0.026	−			
[6] BSI-Anxiety	0.62	0.54	0.055	0.131	−0.020	0.028	0.623**	−		
[7] BSI- Obsession-compulsion	1.06	0.73	0.114	0.079	−0.027	0.011	0.653**	0.603**	−	
[8] ACSID-11 Frequency^a^	15.0	8.02	0.089	0.030	−0.004	0.019	0.268**	0.282**	0.449**	−
[9] ACSID-11 Intensity^a^	13.4	7.40	0.126	0.018	0.020	0.015	0.285**	0.271**	0.407**	0.894**
[10] FTND score^b^	6.44	1.31	−0.032	−0.223	0.199	−0.204	−0.127	0.274	−0.328	−c

* *p* < 0.05, ** *p* < 0.01.

*Note*. IAT = Implicit Association Test, AAT = Approach-Avoidance task, BSI = Brief Symptom Inventory, ACSID-11 = Assessment of Specific Criteria for Internet-use disorders, FTND = Fagerström Test for Nicotine Dependence.

a Correlations with ACSID-11 scores could only be calculated for the subsample of individuals using social networks (n = 162).

b Correlations with FTND scores could only be calculated for the subsample of individuals with TUD (n = 16).

c Due to the different subsamples, no correlations could be calculated between FTND and ACSID-11 scores.

### 4.2 Group comparisons based on four groups

Table 3 illustrates the comparisons of measures of implicit cognitions between all groups. In a first step, we tested H1 regarding the relevance of implicit associations in the different groups. Results regarding the D2_SD_ score of the IAT showed significant differences when comparing all four groups illustrating a small to medium effect. Bonferroni post-hoc analysis revealed a significant difference between the TUD group showing the lowest expressions and the risky group (*p* = 0.040) and the PSNU group (*p* = 0.038), both showing the highest. However, when controlling for age, depression, anxiety, and obsession/compulsion as covariates, the group differences in the D2_SD_ score did not remain significant in subsequent ANCOVA-analysis (*F*(3, 170) = 2.25, *p* = 0.084, *η^2^* = 0.04).

**Table 3:** Descriptive Statistics and comparisons of measures of implicit cognitions between groups.

			**SNUD groups**						**TUD** **(*n* = 16)**		**4-group-comparison**						**3-group-comparison**					
**Measure**	**Overall**		**PSNU** **(*n* = 53)**		**Risky** **(*n* = 61)**		**control** **(*n* = 48)**															
	*Mean*	*SD*	*Mean*	*SD*	*Mean*	*SD*	*Mean*	*SD*	*Mean*	*SD*	Test	*F*	*df*1	*df*2	*p*	*η*2	Test	*F*	*df*1	*df*2	*p*	η2
*IAT Score*																						
D2_SD_	0.70	0.52	0.77	0.47	0.76	0.51	0.65	0.55	0.37	0.51	ANOVA	3.03	3	174	0.031	0.05	ANOVA	0.86	2	159	0.424	0.01

*AAT Scores*																						
Target compatibility effect Score	−128.44	88.60	−128.78	94.87	−116.10	93.62	−135.45	62.71	−153.38	112.25	ANOVA	0.92	3	174	0.434	0.02	ANOVA	0.72	2	159	0.488	0.01
Neutral compatibility effect Score	−184.86	109.98	−204.80	141.73	−165.76	97.99	−182.02	81.28	−200.09	104.02	ANOVA	1.31	3	174	0.271	0.02	ANOVA	1.78	2	159	0.171	0.02
Overall RT Score	−34.93	41.86	−29.66	43.09	−42.93	44.55	−30.72	33.26	−34.50	49.17	ANOVA	1.19	3	174	0.316	0.02	ANOVA	1.85	2	159	0.161	0.02

*Note*. PSNU = problematic use of social networks; TUD = tobacco use disorder; IAT = Implicit Association Test, AAT = Approach-Avoidance task.

Secondly, we tested the H2 to identify potential group differences in the AAT scores. The results revealed no significant group differences for the target compatibility effect score, the neutral compatibility effect score, nor the overall-RT score. There were no changes for the target compatibility effect score (*F*(3, 170) = 0.74, *p* = 0.533, *η^2^* = 0.01), neutral compatibility effect score (*F*(3, 170) = 1.39, *p* = 0.249, *η^2^* = 0.02), and overall-RT score (*F*(3, 170) = 1.11, *p* = 0.345, *η^2^* = 0.02) when controlling for the covariates mentioned.

### 4.3 Group comparisons based on three groups

Additional analyses (see Table 3) were performed to enable a better comparison of the three groups of social network users (PSNU group, risky group, and control group) omitting the low statistical power of the group of individuals with TUD. The respective ANOVA showed no significant group differences regarding the D2_SD_ score of the IAT. Again, when controlling the D2_SD_ score for age and BSI-subscales of depression, anxiety and obsession/compulsion no significant group differences between the three groups occurred (*F*(2,155) = 0.61, *p* = 0.546, *η^2^* = 0.01). Also, a three-group analysis based on the AAT scores revealed no further group differences for target compatibility effect score, neutral compatibility effect score and Overall-RT score. Controlling for psychopathology and age also revealed no additional effects for target compatibility effect score (*F(*2,155) = 1.19, *p* = 0.307, *η^2^* = 0.02), neutral compatibility effect score (*F*(2,155) = 1.79, *p* = 0.170, *η^2^* = 0.02) and overall-RT score (*F*(2,155) = 1.71, *p* = 0.184, *η^2^* = 0.02).

### 4.4 Exploratory analyses

Additional exploratory analyses investigating the relation between ACSID-11 scores and the AAT compatibility effect scores for target stimuli in the subsample of the social network groups revealed no significant linear relationship (*F*(1,160) = 0.15, *p* = 0.701, *R^2^* = 0.001, β_1_ = 0.003) or quadratic relationship (*F*(2,159) = 0.284, *p* = 0.753, *R^2^* = 0.004, β_1_ = -0.004, β_2_ = -3.19x10^-5^) for the frequency score. No further linear (*F*(1,160) = 0.05, *p* = 0.824, *R^2^* < 0.001, β_1_ = 0.002) or quadratic (*F*(2,159) = 0.225, *p* = 0.799, *R^2^* = 0.003, β_1_ = -0.005, β_2_ = -2.877x10^-5^) association was detected regarding the intensity score.

## 5 Discussion

The present study investigated implicit associations and approach-avoidance tendencies toward addiction-related stimuli in individuals with PSNU, risky social network use, non-problematic use, and TUD. Contrary to our hypotheses, individuals with PSNU and TUD did not show stronger implicit associations or approach tendencies compared to the other groups. Although individuals with PSNU and risky use initially exhibited more positive implicit associations than individuals with TUD, these effects did not remain significant after controlling for age and psychopathological symptoms. No group differences were observed for approach-avoidance tendencies, and exploratory analyses revealed neither linear nor quadratic associations with PSNU symptom severity.

Regarding implicit associations, previous research generally reports, individuals with TUD to show stronger positive implicit associations towards addiction-related stimuli than non-smokers, although findings vary depending on methodological characteristics and the applied stimuli (Gao et al., 2022, Glock and Pit Ten-Cate, 2019, Sherman et al., 2003). In particular, some authors argue in line with the incentive-sensitization theory, that valence-based IATs may preferentially capture hedonic evaluations (“liking”) rather than incentive salience (“wanting”), which has been proposed as a central motivational mechanism in TUD (Grigutsch et al., 2019). This distinction may partly explain the comparatively lower IAT scores in the TUD group.

Concerning PSNU, our present findings add to an increasingly heterogeneous literature. While some studies have reported positive associations between implicit attitudes and PSNU symptom severity (Brailovskaia and Teichert, 2020, Turel and Serenko, 2020), other studies failed to identify clear group differences (Kessling et al., 2025). One possible explanation could be that the use of social networks is strongly driven by rewarding and socially reinforcing experiences that occur across different stages of usage (Brand et al., 2025, Wegmann et al., 2025). As implicit cognitions are shaped by repeated experience and conditioning processes (Stacy & Wiers, 2010), positive implicit associations toward social network stimuli may already be present in non-problematic and especially risky stages of usage. The latter is also supported by findings on other behavioral addictions showing that positive implicit associations towards behavior related stimuli are often already detected in subclinical samples (e.g. Snagowski et al., 2015). The findings that group differences did not persist after controlling for depressive and obsessive–compulsive symptoms further suggests that general psychopathological burden may modulate implicit associations in PSNU, as it has already been demonstrated for substance use disorders (Cohn et al., 2014).

With regard to the approach-avoidance tendencies, our present findings revealed no group differences between individuals with PSNU, risky use, non-problematic use, and TUD. These results contrast with previous findings in substance use disorders suggesting stronger approach tendencies toward addiction-related stimuli in clinical samples (Kakoschke et al., 2019, Wiers et al., 2013), although some studies also reported null effects or even avoidance of addiction-related stimuli (Larsen et al., 2014). Previous findings specifically in PSNU were mixed. While Juergensen and Leckfor (2019) reported a positive association between approach tendencies and PSNU symptom severity, another study found approach tendencies to be linked to usage frequency in general rather than problematic use (Wadsley & Ihssen, 2022). Notably, in our present study, addiction-related stimuli were rather avoided across all subgroups, as indicated by negative mean compatibility effect scores. Against this background, it might be important, as outlined by Breiner et al. (1999) to more systematically consider behavior-specific outcome expectancies, craving experiences, as well situational factors, in order to determine whether approach-avoidance tendencies in certain situations predict individual’s impulses to conduct a specific behavior despite long-term negative consequences. Some researchers already argued that the occurrence of addictive behaviors may best be described by an evaluative space in an addiction-related decision situation, which can be divided into states of approach, avoidance, ambivalence, and indifference (Breiner et al., 1999, Snagowski and Brand, 2015). Given that, these additional factors might explain why approach tendencies predict problematic behavior only under certain circumstances.

From a theoretical perspective, the present findings can be interpreted within the I-PACE framework (Brand et al., 2019, Brand et al., 2025), which assumes that addictive behaviors arise from dynamic interactions between person-related variables, affective responses, and cognitive processes. In line with this framework, our results suggest implicit cognitive processes such as automatic associations and approach tendencies not to be considered as stable trait-like characteristics but merely as dynamic processes, maybe influenced by several further factors. For example, individual motivational states may highly influence the occurrence of these mechanisms. Studies from substance use disorders have already shown the individual level of deprivation to influence cue-reactivity, which in turn may alter implicit associations (Tibboel et al., 2024). Moreover, differences outcome expectancies may further explain variance in implicit associations and approach avoidance tendencies, as these expectancies been shown to be further vary according to factors like boredom and stress (Wegmann, Ostendorf, & Brand, 2018). That said, it could be hypothesized whether individuals with a high symptom burden and a high perceived loss of control might also develop negative expectations of utility, which in turn could lead to a weakening in implicit cognitions, which has already been discussed for approach-avoidance tendencies in alcohol use disorder (Townshend & Duka, 2007). Moreover, studies from the field of alcohol use disorder already found moderating effects for working memory capacity and response inhibition (Lavigne et al., 2017, Rowland et al., 2021). The I-PACE model also emphasizes generally diminished cognitive control in later stages of the addictive process (Brand et al., 2019, Brand et al., 2025), interindividual differences in inhibitory control and executive functions may still exist and may therefore account for further variance in in implicit associations and approach avoidance tendencies.

Taken together, the present findings suggest that implicit cognitive processes play a more nuanced role in PSNU and might therefore not be suitable stable markers for problematic usage. We suggest a more dynamical interpretation of dual-process models that address the approach of a more complex interplay of impulsive tendencies and reflective control, craving, use expectancies, and predisposing factors. Direct comparisons between PSNU and substance use disorders such as TUD might still be useful to identify overlapping and distinct mechanisms. These comparisons should further incorporate additional variables, as differences may exist regarding outcome-expectancies, general stress und the subjective experience of impairment and psychological distress (Moretta & Wegmann, 2025).

### 5.1 Limitations and strengths

Several limitations should be acknowledged. The small sample of the TUD group limits statistical power. Recruiting individuals with TUD who simultaneously show non-problematic use of social networks according to our criteria proved challenging (see also Montag et al., 2023), reflecting the high comorbidity rates between these two behaviors which have already been described in previous works (Buja et al., 2018, Chiao et al., 2014). Second, the stimulus material used in the implicit tasks was only semi-individualized and may not have fully captured participants’ usage reality. However, the same stimulus material has been applied in previous studies and has shown to induce robust craving reactions in individuals (Antons et al., 2025, Wegmann et al., 2021). Especially in the IAT procedure, addiction-related stimuli of the PSNU group served as control stimuli for the TUD group and vice versa, which may have caused biases if these stimuli were not neutrally associated. However, it must be noted that the comparison with objectively neutral stimuli in the AAT did not reveal any particular effects. Third, laboratory-based implicit IATs and AATs have been criticized by some researchers regarding construct validity (Teige-Mocigemba et al., 2010) and especially ecological validity, as they may not adequately reflect real-life decision situations (Fricke & Vogel, 2020). Against this background, we find the implementation of mobile measures, e.g., smartphone-based (Zech et al., 2023) or virtual-reality based AATs (Wei et al., 2023) that are already implemented in other research fields, a promising approach for future research. Nevertheless, to our knowledge, our study is the first to investigate the role of implicit associations and approach-avoidance tendencies together in PSNU by drawing a direct comparison towards a substance use disorder, while simultaneously relying on data derived from diagnostic interviews.

## 6 Conclusions and future directions

In conclusion, the present study does not support the assumption that individuals with PSNU or TUD consistently exhibit stronger positive implicit associations or approach tendencies toward addiction-related stimuli. Our findings highlight substantial heterogeneity in implicit cognitive processes in PSNU and suggest that these may already emerge in early stages of PSNU and might be subsequent to predisposing individual characteristics and situational factors. Future research should adopt more dynamic and ecologically valid approaches to investigate interactions between implicit cognitions, affective states, craving, and situational influences in PSNU. This may further inform the development and more targeted application of therapeutic approaches such as cognitive bias modification by emphasizing that implicit cognitive processes vary as a function of individual characteristics and situational factors.

## Ethics

All procedures were carried out in accordance with the Declaration of Helsinki. The ethics committees of the University of Lübeck (ID: 20-081) and the University of Duisburg-Essen (ID of overall project: 1911APBM0457) approved the study procedure.

## Open science

This sub-project and all its hypotheses were pre-registered on Open Science Framework (https://osf.io/9j8g3/).

## CRediT authorship contribution statement

**Lasse David Schmidt:** Writing – original draft, Project administration, Methodology, Investigation, Formal analysis, Data curation. **Lena Klein:** Writing – review & editing, Software, Methodology, Investigation, Data curation. **Christian Montag:** Writing – review & editing, Methodology, Funding acquisition, Conceptualization. **Hans-Jürgen Rumpf:** Writing – review & editing, Funding acquisition, Conceptualization. **Elisa Wegmann:** Writing – review & editing, Supervision, Methodology, Funding acquisition, Conceptualization.

## Funding

The procedure presented was carried out as part of a sub-project of the FOR2974 research group (Preregistration of research group: https://osf.io/n5cd7/), funded by the German Research Foundation (DFG; grant ID: 411232260), at the universities of Lübeck and Duisburg-Essen.

## Declaration of competing interest

The authors declare that they have no known competing financial interests or personal relationships that could have appeared to influence the work reported in this paper.

## Data Availability

Data will be stored in OSF repository upon publication
